# With or without reirradiation in advanced local recurrent nasopharyngeal carcinoma: a case–control study

**DOI:** 10.1186/s12885-016-2803-2

**Published:** 2016-10-07

**Authors:** Li-Ting Liu, Qiu-Yan Chen, Lin-Quan Tang, Lu Zhang, Shan-Shan Guo, Ling Guo, Hao-Yuan Mo, Chong Zhao, Xiang Guo, Ming-Yuan Chen, Chao-Nan Qian, Mu-Sheng Zeng, Ming-Huang Hong, Jian-Yong Shao, Ying Sun, Jun Ma, Hai-Qiang Mai

**Affiliations:** 1 State Key Laboratory of Oncology in South China; Collaborative Innovation Center for Cancer Medicine, Sun Yat-sen University Cancer Center, Guangzhou, 510060 China; 2 Department of Nasopharyngeal Carcinoma, Sun Yat-sen University Cancer Center, 651 Dongfeng Road East, Guangzhou, 510060 China; 3 Good Clinial Practice Center, Sun Yat-sen University Cancer Center, Guangzhou, 510060 People’s Republic of China; 4 Department of Molecular Diagnostics, Sun Yat-sen University Cancer Center, Guangzhou, 510060 China; 5 Department of Radiation Oncology, Sun Yat-sen University Cancer Center, Guangzhou, 510060 People’s Republic of China

**Keywords:** Recurrent nasopharyngeal carcinoma, Reirradiation, Chemotherapy

## Abstract

**Background:**

The study aimed to evaluate the long-term outcome in patients with advanced local recurrent nasopharyngeal carcinoma (NPC) treated with or without reirradiation.

**Methods:**

A total of 44 patients treated without reirradiation (non-RT + chemotherapy) were matched with 44 patients treated with reirradiation (re-RT+/-chemtherapy) by age, sex, Karnosky performance score (KPS), rT stage, rN stage, and time interval between initial radiation and recurrence (TI). Overall survival (OS) rate and time to progression (TTP) rate were assessed using Kaplan–Meier method, log-rank test, and Cox regression analysis.

**Results:**

From March 2008 to December 2013, a total of 88 well-balanced rT3–4 N0-1 NPC patients were retrospectively analyzed. After a median follow-up of 27 months (range: 6–85), the 5-year OS rate and TTP rate was 23.4 %, 39.0 % in the non-RT + chemotherapy group and 27.5 %, 49.8 % in the re-RT+/-chemtherapy group, respectively. Multivariate analysis showed that significant toxic effect was the only significant prognosticator correlated with OS (HR: 2.15, 95 % CI = 1.02–4.53, *p* = 0.044). No statistically significant survival differences were observed between the two treatment groups in either univariate or multivariate analyses.

**Conclusion:**

Compared with reiradiation, treating advanced local recurrent NPC with chemotherapy alone warrants further validation in the view of its similar survival and more acceptable toxicities.

**Electronic supplementary material:**

The online version of this article (doi:10.1186/s12885-016-2803-2) contains supplementary material, which is available to authorized users.

## Background

Nasopharyngeal carcinoma (NPC) is the predominant malignancy arising from the nasopharynx epithelium. Radiation therapy (RT) is the primary treatment for NPC due to its radiosensitive behavior and deep-seated anatomic location. For advanced-stage NPC, concurrent chemoradiotherapy (CCRT) is the established standard treatment protocol [1–6]. With the development of modern radiation and imaging techniques, the local control rate of NPC has been favorably improved. However, 10–15 % of patients still experience local recurrence [7–9]. A second course of RT with the technique of conventional external beam radiotherapy (EBRT), brachytherapy, and stereotactic radiosurgery were commonly used as salvage treatment in the past decades. Nonetheless, both the results of tumor control and the patient’s quality of life are barely satisfactory [10–12]. The advent of intensity modulated radiation therapy (IMRT) brought improvement of target coverage and the sparing of adjacent critical organs [13]. With IMRT, reirradiation for local recurrent NPC achieved encouraging tumor control and patient survival; further, the treatment-related toxicities were acceptable [14–17]. However, when we went deep into these results, we found that reirradiation mainly prolongs survival of patients with early local recurrent stage disease. The effect of reirradiation in the treatment of advanced local recurrent disease remains uncertain. Moreover, patients with advanced local failure invariably experienced excessive risk of severe late complications of reirradiation caused by extensive tumor infiltration of critical normal tissues in the vicinity of tumor target that had already received a high dose of radiation from the primary RT. In view of these potential RT-related severe toxicities, quite a few patients with advanced rT stage disease refused to take the second course of RT. Then chemotherapy became the alternative treatment method for these patients. However, chemotherapy has been considered a palliative therapy in the salvage treatment of local recurrent disease [18, 19]. In fact, the efficacy of chemotherapy in treating advanced local recurrent NPC has not yet been fully evaluated. In our daily clinical work, survival of these patients was observed to approach the rate of patients treated with RT. Thus, the aim of this case-control study is to assess survival of patients with advanced local recurrent NPC treated with or without reirradiation. We hypothesize that patients with advanced local failure treated with chemotherapy alone (non-RT + chemotherapy) have a survival rate equivalent to patients treated with RT with or without chemotherapy (re-RT+/-chemtherapy) but with fewer treatment-related complications.

## Methods

Patients diagnosed with advanced local recurrent NPC between March 2008 and December 2013 in our institute were identified. The eligibility criteria included the following: (1) rT3-4 N0-1 disease according to the 7^th^ edition of the International Union against Cancer/American Joint Committee on Cancer (UICC/AJCC) staging system; (2) with retropharyngeal lymph node metastasis only; (3) no evidence of distant metastases; (4) aged 18 years or older; (5) absence of secondary malignancy, pregnancy or lactation; (6) adequate hematologic function (white blood cell counts ≥ 4000/μL and platelet counts ≥ 100000/μL), adequate renal function (creatinine clearance ≥ 50 mL/min) and adequate hepatic function (serum bilirubin level < 1.5 mg/dL) before treatment, and (7) treated with IMRT+/-chemotherapy or chemotherapy alone.

Patients who met the inclusion criteria were divided into two groups: non-RT + chemotherapy group and re-RT+/-chemotherapy group. For comparisons, the 44 recurrent patients in the non-RT + chemotherapy group were individually matched to one control patient in the re-RT+/-chemotherapy group according to age, sex, Karnosky performance score (KPS), rT stage, rN stage, and time interval between initial radiation and recurrence (TI). Local recurrence of most patients was proved by biopsy. Patients with recurrence in inaccessible sites, such as the cavernous sinus and skull base, were diagnosed according to their clinical symptoms and image manifestations. All of the patients were retrospectively re-staged according to the seventh edition of the International Union Against Cancer/American Joint Committee on Cancer (UICC/AJCC) staging system.

### Pretreatment evaluation

All patients were evaluated through a complete physical examination, fiber-optic nasopharyngoscopy, and complete blood sampling, including differential cell counts, biochemical profile, and plasma level of EBV DNA measured by real-time quantitative polymerase chain reaction (PCR) [20, 21] before treatment. Magnetic resonance imaging (MRI) of the nasopharynx and neck, chest X-ray, abdominal sonography, electrocardiography, and bone scan, or 18 F-FDG positron emission tomography (PET)/computed tomography scans were carried out for accurate disease staging. This retrospective study was approved by the Clinical Research Committee of the study institute.

### Chemotherapy

Cisplatin-based chemotherapy (cisplatin alone or cisplatin plus other one or two anti-tumor drugs, including 5-fluorouracil, paclitaxel and gemcitabine) was administered to 37 patients treated with RT and 44 patients without RT. In the re-RT+/-chemtherapy group, eight patients underwent concurrent chemotherapy, 17 patients underwent induction chemotherapy, and 12 patients underwent both induction and concurrent chemotherapy [18, 19, 22, 23]. In the non-RT + chemtherapy group, after undergoing two to six cycles of chemotherapy, oral tegafur-uracil or capecitabine was administered to 29 patients for maintenance chemotherapy until disease progression or death.

### Radiation therapy

All of the patients in the re-RT+/-chemotherapy group used IMRT. The IMRT plan was designed according to the treatment protocol for recurrent NPC at our study institute. All patients were immobilized in the supine position with a head, neck, and shoulder thermoplastic mask. Two sets of images, with and without contrast, were obtained from the CT simulator for treatment planning. All patients were scanned with serial 3 mm slices from the vertex through the clavicles. The imaging data were transferred to the Corvus inverse planning system (Peacock, Nomos, Deer Park, IL), and a MiMi multileaf collimator (Nomos, Sewickly, PA) was used for planning and treatment.

Tumor volumes were delineated in accordance with the International Commission on Radiation Units and Measurements Report 62 (ICRU 62) and ICRU 50. The delineation of recurrent gross tumor volumes (GTVnx and GTVnd) was based on the MRI. The clinical tumor volume (CTV) included GTV plus a 5 to 10 mm margin and encompassed the recurrent lymph node. Critical normal structures, including the brainstem, spinal cord, parotid glands, optic nerves, chiasm, lens, eyeballs, temporal lobes, temporomandibular joints, mandible, and hypophysis were contoured and set as organ at risk (OARs) during optimization. The planning target volume (PTV) was created based on each volume, with an additional 3 mm margin, allowing for setup variability.

The prescribed dose was 58–70 Gy to the GTV and 50–54 Gy to the CTV in 27 to 35 fractions. The dose–volume histograms of the treatment targets and critical normal structures were evaluated. For GTV and CTV, the target volumes receiving 95 % of the prescribed dose were used to reflect the target coverage. The dose constraints to the critical organs were limited by the threshold doses, the TI after primary RT, and the patient’s performance status.

### Outcome and follow-up

The primary endpoint for the study was overall survival (OS), defined as the time from the day of therapy to the date of death from any cause or patient censoring at the date of the final follow-up. The secondary endpoints for the study were toxic effects and time to progression (TTP), which was defined as the time to date of treatment failure at any site or patient censoring at the date of the last follow-up. After the completion of treatment, patients were examined at least every 3 months during the first 3 years and had follow-up examinations every 6 months thereafter or until death. Nasopharyngoscopy, MRI of the head and neck, chest radiography, abdominal sonography, or PET-CT were routinely performed annually or upon clinical indication of tumor relapse. Acute toxicities were classified according to the Common Toxicity Criteria (CTC) system version 3.0 and were assessed weekly during retreatment. For patients in the RT group, the most severe radiation-related toxicities were assessed and graded based on the Radiation Therapy Oncology Group (RTOG)/European Organization for Research and Treatment of Cancer (EORTC) morbidity scoring system.

### Statistical analysis

Statistical analyses were performed using SPSS software (version 20.0, SPSS Inc., Chicago, IL, USA). Fisher’s exact test and a *χ*2 test were used to assess categorical variables, whereas the *t*-test and Mann–Whitney *U* test were used to analyze continuous variables. The actuarial survival rates were estimated by the Kaplan–Meier method and survival curves were compared using the log-rank test. Univariate and multivariate analyses were performed using the Cox proportional hazards model. The following factors were included in the univariate and multivariate analyses: treatment methods (re-RT+/-chemotherapy or non-RT + chemtherapy), age, sex, rT stage, rN stage, EBV DNA (=0 copies per milliliter vs. > 0 copies per milliliter), KPS, TI, and significant toxic effects (Grade3–5 treatment-related late toxicity). All statistical tests were two-sided, and a *P* value of less than 0.05 was considered significant.

## Results

The patient characteristics are detailed in Table 1. The re-RT+/-chemotherapy group and non-RT + chemtherapy group each had 44 patients. The groups were well matched for age, sex, KPS, rT stage, rN stage, and TI.

**Table 1:** Patient characteristics

	Re-RT+/-chemothrapy group	Non-RT + chemtherapy group	
Characteristic	No. of patients (%)	No. of patients (%)	*P* value
Total	44	44	
Age, y			0.286
Median	45	48	
Range	29–62	30–63	
Sex			1.000
Female	10 (22.7)	10 (22.7)	
Male	34 (77.3)	34 (77.3)	
Pathology			0.597
WHO type 2–3	36 (81.8)	34 (77.3)	
Imaging manifestation only	8 (18.2)	10 (22.7)	
rT stage			1.000
T3	20 (45.6)	20 (45.6)	
T4	24 (54.5)	24 (54.5)	
rN stage			1.000
N0	33 (75.0)	33 (75.0)	
N1	11 (25.0)	11 (25.0)	
TI (months)			1.000
> 24	20 (45.6)	20 (45.6)	
≤ 24	24 (54.5)	24 (54.5)	
Chemotherapy			0.012
Yes	37 (84.1)	44 (100)	
No	7 (15.9)	0 (0)	
KPS			1.000
> 70	40 (90.9)	40 (90.9)	
≤ 70	4 (9.1)	4 (9.1)	
Significant toxic effects			<0.001
Yes	38 (86.4)	23 (52.3)	
No	6 (13.6)	21 (47.7)	

*Abbreviations: RT* radiation therapy, *TI* time interval between initial radiation and recurrence, *KPS* Karnosky performance score, *Significant toxic effects* Grade 3–5 treatment-related late toxicity, *Re-RT+/-chemotherapy* radiation therapy with or without chemotherapy, *non-RT + chemotherapy* chemotherapy alone

### RT treatment plans

The median minimum, mean, and maximum GTVnx doses given to the 44 patients were 62.3 Gy (range, 53.9–73.5 Gy), 66.3 Gy (range, 58.6–77.0 Gy), and 71.2 Gy (range, 61.5–80.5 Gy), respectively. The median volume of GTVnx was 54.5 cm^3^ (range, 15.2–121.8 cm3).

### Tumor response assessment

Three months after completion of therapy, the response of all patients was evaluated by the investigator according to the Response Evaluation Criteria in Solid Tumors (RECIST) criteria [24] as complete response (CR), partial response (PR), stable disease (SD), disease progression (PD), or not assessable. If the recurrent disease exhibited a CR or a PR to treatment, we considered the patient to be a responder. The response rate in the non-RT + chemtherapy group was 52.3 %: 5 patients exhibited CR, 18 patients exhibited PR, 17 patients exhibited SD, and 4 patients exhibited PD. The response rate in the re-RT+/-chemtherapy group was 79.5 %: 12 patients exhibited CR, 23 patients exhibited PR, 8 patients exhibited SD, and 1 patient exhibited PD.

### Toxicities

In the re-RT+/-chemtherapy group, different grades of acute and late toxicities were observed in all patients (Table 2). The most common acute toxicities included Grade 1 to 2 mucositis and xerostomia. Eight (18.2 %) patients suffered from Grade 3 mucositis, 9 (20.5 %) patients suffered from Grade 3 xerostomia, and 2 (5 %) patients suffered from Grade 4 mucositis. Grade 3 to 4 anemia, neutropenia, and thrombocytopenia were encountered in 11 (25.0 %), 16 (36.4 %), and 14 (31.8 %) patients, respectively. After completion of reirradiation, 17 (38.6 %) patients experienced nasopharyngeal necrosis, 8 (18.2 %) patients experienced temporal lobe necrosis, 6 (13.6 %) patients experienced cranial neuropathy, 21 (47.7 %) patients experienced hearing loss, and 9 (20.5 %) patients experienced trismus. Grade 3 to 5 late toxicities were 23 (52.3 %) in the re-RT+/-chemtherapy group during the follow-up period. In the non-RT + chemtherapy group, hematological toxicity was the major toxicity observed in the treatment course. Grade 3 to 4 anemia, neutropenia, and thrombocytopenia were encountered in 12 (27.3 %), 19 (43.2 %), and 13 (29.5 %) patients. Only one (2.2 %) patient with neutropenic sepsis was documented. Grade 1 to 2 radiation-related toxicity from the primary RT, including xerostomia, hearing loss, trismus, and temporal lobe necrosis, was observed in the patients in the control group. Only five (11.4 %) patients developed Grade 3 radiation-related toxicity (2 with xerostomia, 2 with hearing loss, and 1 with temporal lobe necrosis).

**Table 2:** Treatment related toxicities

	RT+/-chemotherapy group	non-RT + chemotherapy group	
	No.of patients (%)	No.of patients (%)	*P* value
Grade3-5 Anemia			0.808
No	33 (75.0 %)	32 (72.7 %)	
Yes	11 (25.0 %)	12 (27.3 %)	
Grade3-5 Neutropenia			0.513
No	28 (63.6 %)	25 (56.8 %)	
Yes	16 (36.4 %)	19 (43.2 %)	
Grade3-5 Thrombocytopenia			0.817
No	30 (68.2 %)	31 (70.5 %)	
Yes	14 (31.8 %)	13 (29.5 %)	
Grade3-5 Mucositis			0.001
No	34 (77.3 %)	44 (100.0 %)	
Yes	10 (22.7 %)	0 (0 %)	
Grade3-5 Xerostomia			0.053
No	35 (79.5 %)	42 (95.5 %)	
Yes	9 (20.5 %)	2 (4.5 %)	
Grade3-5 Nasopharyngeal necrosis			<0.001
No	27 (61.4 %)	44 (100.0 %)	
Yes	17 (38.6 %)	0 (0 %)	
Grade3-5 Temporal lobe necrosis			0.035
No	36 (81.8 %)	43 (97.7 %)	
Yes	8 (18.2 %)	1 (2.3 %)	
Grade3-5 Cranial neuropathy			0.026
No	38 (86.4 %)	44 (100.0 %)	
Yes	6 (13.6 %)	0 (0 %)	
Grade3-5 Trismus			
No	35 (79.5 %)	44 (100.0 %)	0.002
Yes	9 (20.5 %)	0 (0 %)	
Grade3-5 Hearing loss			0.001
No	28 (63.6 %)	42 (95.5 %)	
Yes	16 (36.4 %)	2 (4.5 %)	

*Abbreviations*: *RT* radiation therapy, *Re-RT+/-chemotherapy* radiation therapy with or without chemotherapy, *non-RT + chemotherapy* chemotherapy alone

### Survival

**Fig. 1:**
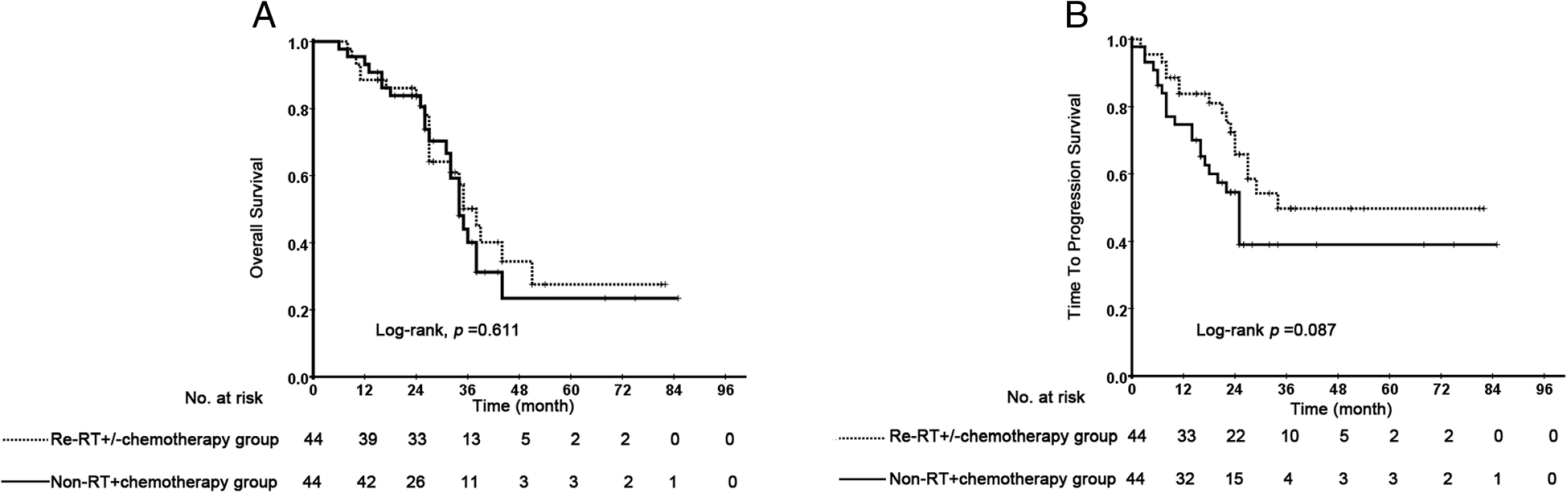
Kaplan–Meier curves of overall survival (**a**) and time to progression survival (**b**) in advanced local recurrent NPC patients treated with re-RT+/-chemtherapy and non-RT + chemotherapy

Within the median follow-up duration of 27 months (range, 6–85 months), 34 developed local failure, 11 exhibited distant metastasis, and 44 patients died. In the re-RT+/-chemtherapy group, 13 patients died due to radiation-related injuries (3 from radiation encephalopathy, 8 patients from mucosal necrosis or massive hemorrhage, and 2 patients from other radiation-related injuries), 4 patients died due to local failures, 3 patients died due to distant metastasis, 1 patient died due to pneumonia, and 1 died due to intracranial infection (Table 3). In the non-RT + chemtherapy group, 13 patients died due to progression of local disease, 3 patients died due to distant metastasis, 5 patients died due to unknown cause (Their family members refused to tell the cause of death) and 1 patient died due to neutropenic sepsis (Table 3). The 5-year overall survival rate was 23.4 % (95 % CI = 4.6 %–42.2 %) in the non-RT + chemtherapy group and 27.5 % (95 % CI = 8.1 %–46.9 %) in the re-RT+/-chemtherapy group (*p* = 0.611) (Fig. 1). No statistically significant survival differences were observed between the two groups. The 5-year TTP rate was 39.0 % (95 % CI = 22.1 %–55.9 %) in the non-RT + chemtherapy group and 49.8 % (95 % CI = 31.8 %–67.8 %) in the re-RT+/-chemtherapy group (*p* = 0.087) (Fig. 1). Although the non-RT + chemtherapy group had about a 10 % higher risk of disease progression than the re-RT+/-chemtherapy group, the difference was not significant. Univariate analyses revealed that KPS (HR: 3.59, 95 % CI = 1.56–8.30, *p* = 0.003) and significant toxic effects (HR: 1.98, 95 % CI = 1.09–3.59, *p* = 0.025) were significantly correlated with OS. Univariate analysis also demonstrated that KPS (HR: 2.49, 95 % CI = 1.04–5.95, *p* = 0.041) was significantly associated with TTP. Multivariate analyses were performed to adjust further for various prognostic factors, including age (>46 years vs. ≤46 years), sex (female vs. male), rT stage (rT3 vs. rT4), rN stage (rN0 vs. rN1), EBV DNA (= 0 copies per milliliter vs. > 0 copies per milliliter), TI (> 24 month vs. ≤ 24 month), KPS (> 70 vs. ≤ 70), significant toxic effects (Grade 0–2 treatment-related late toxicity vs. Grade 3–5 treatment-related late toxicity) and treatment methods (re-RT+/-chemtherapy vs. non-RT + chemtherapy). Multivariate analysis revealed that significant toxic effects (HR: 2.12, 95 % CI = 1.01–4.47, *p* = 0.047) was the only significant prognosticator associated with OS (Table 4). Both univariate and multivariate analyses demonstrated that patients treated with non-RT + chemtherapy methods were not associated with higher risk of death and disease progression than patients treated with re-RT+/-chemtherapy methods.

**Table 3:** Cause of death of the 88 patients with advanced local recurrent NPC

	Aggressive treatment group	Conservative treatment group
Death	No. of patients (%)	No. of patients (%)
Total	22	22
Local failure	4(18.2)	13(59.1)
Distant metastasis	3(13.6)	3(13.6)
Radiation injuries	13(59.1)	0(0)
Others	2(9.1)	6(27.3)

*Abbreviations: NPC* nasopharyngeal carcinoma, *RT* radiation therapy, *Re-RT+/-chemotherapy* radiation therapy with or without chemotherapy, *non-RT + chemotherapy* chemotherapy alone

**Table 4:** Multivariate analysis of prognostic factors correlated with outcome

Endpoint	HR (95 % CI)	*P* value
OS		
Age	0.91 (0.46–1.81)	0.785
Sex	1.05 (0.45–2.45)	0.920
rT stage	1.12 (0.60–2.35)	0.618
rN stage	0.86 (0.40–1.86)	0.701
EBV DNA	0.67 (0.34–1.32)	0.241
KPS	2.36 (0.90–6.12)	0.082
TI	0.89 (0.46–1.72)	0.730
Significant toxic effects	2.12 (1.01–4.47)	0.047
Treatment method	0.61 (0.31–1.21)	0.158
TTP		
Age	0.60 (0.28–1.29)	0.191
Sex	0.90 (0.43–1.91)	0.784
rT stage	1.20 (0.60–2.39)	0.611
rN stage	1.13 (0.53–2.43)	0.750
EBV DNA	1.55 (0.73–3.29)	0.251
KPS	2.10 (0.76–5.81)	0.155
TI	0.89 (0.46–1.73)	0.730
Significant toxic effects	1.21 (0.55–2.67)	0.640
Treatment method	0.49 (0.24–1.02)	0.056

*Abbreviations: OS* overall survival, *TTP* time to progression, *TI* time interval between initial radiation and recurrence, *KPS* Karnosky performance score, *Significant toxic effects* Grade3–5 treatment-related late toxicity, *Treatment method* patients treated with a radiation therapy with or without chemotherapy or chemotherapy alone

## Discussion

Management of local recurrent NPC is still a crucial clinical challenge, especially to patients with advanced local recurrent disease. Salvage treatment with reirradiation has usually been recommended for improving the long-term survival. However, a second course of RT always comes with severe complications. The 5-year survival rates remain unsatisfactory at about 7 % to 37.0 % [10–12, 25–27]. As IMRT emerges, with favorable dose distribution to the tumor target and adjacent critical organs, it has shown a lot of advantages compared with conventional RT [13, 28]. Kwok et al. reported a large series on patients with recurrent NPC who received RT. The 3-year OS rate for patients with isolated local failure was 74 %. However, in subgroup analysis, salvage treatment was associated with improved OS only in patients with rT1 to rT2 local failure, but not with rT3 or rT4 disease. Hua et al. reported an impressive 80.7 % local control rate of 5 years. Although better local control rate was achieved, the 5-year OS rate was only 38 %, especially for patients with advanced stage disease. Furthermore, the incidence of severe late toxicity happened in 39 % patients with advanced disease [16]. Han et al. analyzed the outcomes of 239 NPC patients with local failure who were reirradiated with IMRT. After the follow-up of 29 months (range: 5–121 months), 120 patients in the study died, and 89 (69.2 %) of them died due to radiation-related injuries [15]. A recent study conducted by Tian et al. stated that the heterogeneity of locally recurrent NPC indicates that not all patients will benefit from reirradiation using IMRT, because they may experience poor disease control and severe late complications [29]. One of the most important heterogeneity problems-influenced curative effect is the recurrent T stage in all these studies. Survival always yielded to severe toxicity in patients with advanced rT stage disease. Consequently, quite a few patients with extensive disease were not willing to take the second course of RT and most of them chose chemotherapy-based treatment instead in our daily clinical work. However, chemotherapy is considered a palliative treatment for advanced recurrence [18, 19]. The study performed by Wong et al. is the first systematic study to explore chemotherapy with or without radiotherapy in patients with recurrent NPC. In this study, the 2-year progression-free survival rates in patients treated with RT is better than in patients treated with chemotherapy alone (58 % vs. 38 %), but the overall survival of the two groups was similar with about 55 % of them alive at 2 years. Despite the 58 % remission rate in the chemoradiotherapy group, patients with extensive local and/or regional failure in the study still tend to do poorly with reirradiation [19]. These unsatisfactory results of chemotherapy could be caused partly by selection bias of suboptimal performance status and patients with extensive disease. Our study is the first case-control study to evaluate the outcome of NPC patients with advanced local failure treated with or without reirradiation. In our study, patients treated with RT could experience relatively better long-term remission tendency compared with patients treated without RT (49.8 % vs. 39.0 %). However, patients in the non-RT + chemtherapy group achieved a 5-year OS rate similar to patients treated with re-RT+/-chemtherapy (23.4 % vs. 27.5 %). In patients with rT3 or rT4 disease, reirradiation has been reported not to be associated with improved OS [30]. The lack of survival benefit of reirradiation may be due to suboptimal dose distribution in the large volume of recurrent diseases compromised with critical organs protection, mortality associated with the radiation-related complications, radiation resistance of the recurrent NPC, or fibroplasias after the primary RT of the nasopharyngeal structures. Treatment toxicity is a crucial consideration in the decision about salvage treatment for local recurrent NPC, especially for those with advanced disease. According to our study, most patients treated without RT suffered from mild to moderate late toxicity. Only one patient died due to treatment-related toxicities. Nevertheless, in the re-RT+/-chemtherapy group, 13 patients died due to radiation-related injuries, and most of them suffered from severe radiation-related complications. The quality of life in these patients is poor. Considering similar survival benefit and more acceptable toxicities, treating carefully selected patients with chemotherapy instead of reirradiation would be feasible. The results of the study might help us choose the optimal treatment method for advanced local recurrent NPC patients in our daily clinical work.

However, there are several limitations of our study. First, the simple size of our study is relatively small, which might make the results of study underpowered. Second, this is a retrospective study in a single center. Besides survival and toxicities, medical cost incurred in an effort to control or alleviate the symptoms of either complication or cancer progression in each group is also an important issues we shoulded concerned. For example, treatment of cranial neuropathy, surgery or debridement of the nasopharyngeal mucusa necrosis and intravenous or oral anti-tumor drugs to control tumor progression do cost a lot. Therefore, a prospective study with emphasis on survival, quality of life measurement and medical cost to control or alleviate the symptoms of either complication or cancer progression is warranted to validate the benefit of chemotherapy without reirradiation in the treatment of advanced local recurrent NPC.

## Conclusion

In conclusion, our study demonstrated that the patients with advanced local recurrent NPC receiving chemothrapy alone without reirradiation achieved equivalent survival compared with patients treated with RT, and the toxicity is more acceptable. It would be feasible to treat advanced local recurrent NPC patients with chemotherapy alone instead of reirradiation. Further investigation is warranted.

## Additional files

Additional file 1: Dataset of included patients. (PDF 24 kb)
Additional file 2: Dataset of included patients. (XLS 42 kb)

## Abbreviations

CR: Complete response
CTC: Common toxicity criteria
CTV: Clinical tumor volume
EBRT: External beam radiotherapy
GTV: Gross tumor volumes
IMRT: Intensity modulated radiation therapy
KPS: Karnosky performance score
MRI: Magnetic resonance imaging
non-RT+chemtherapy: Chemotherapy alone without radiation therapy
NPC: Nasopharyngeal carcinoma
OAR: Organ at risk
OS: Overall survival
PCR: Polymerase chain reaction
PD: Disease progression
PR: Partial response
PTV: Planning target volume
re-RT+/-chemtherapy: Radiation therapy with or without chemotherapy
RT: Radiation therapy
RTOG/ EORTC: Therapy Oncology Group /European Organization for Research and Treatment of Cancer
SD: Stable disease
Significant toxic effects: Grade 3–5 treatment-related late toxicity
TI: Time interval between initial radiation and recurrence
TTP: Time to progression
UICC/AJCC: International Union Against Cancer/American Joint Committee on Cancer
