# Supplementary material for: With or without reirradiation in advanced local recurrent nasopharyngeal carcinoma: a case–control study
Source: BMC Cancer. 2016 Oct 7;16:774. doi: 10.1186/s12885-016-2803-2 (PMC5054630; doi:10.1186/s12885-016-2803-2)
Supplement: Additional file 1: — Dataset of included patients. (PDF 24 kb) [file 12885_2016_2803_MOESM1_ESM.pdf]

ID	Age Range	Pathology	Interva	TI(month)	KPS	EBV DNA	rT stage	rN stage	rM stage
1	21-30	1	7.0	84	60	41500	4	0	0
2	21-30	1	1.0	12	100	0	3	1	0
3	21-30	1	3.3	40	90	0	3	0	0
4	31-40	0	1.6	19	80	183	3	1	0
5	31-40	1	3.2	38	90	0	4	0	0
6	31-40	1	3.5	42	60	9430	4	0	0
7	31-40	1	3.5	42	60	183	4	0	0
8	31-40	1	2.4	29	90	2880	4	0	0
9	31-40	1	2.9	35	80	0	4	0	0
10	31-40	1	6.1	73	90	0	4	0	0
11	31-40	1	2.8	33	90	0	3	0	0
12	31-40	1	1.4	17	80	25500	4	1	0
13	31-40	0	3.0	36	90	3970	4	0	0
14	31-40	0	1.3	15	80	0	4	0	0
15	31-40	1	3.8	45	90	28000	3	1	0
16	31-40	1	1.9	23	80	0	4	0	0
17	31-40	1	2.9	35	90	17600	4	0	0
18	31-40	1	2.1	25	90	1990	4	0	0
19	31-40	1	3.1	37	90	1530	3	1	0
20	31-40	0	2.8	33	90	0	4	0	0
21	31-40	1	1.1	13	70	0	3	0	0
22	31-40	1	8.0	96	90	183	4	0	0
23	41-50	1	1.2	14	90	183	4	1	0
24	41-50	1	1.9	23	90	183	3	0	0
25	41-50	1	1.4	17	90	183	4	0	0
26	41-50	0	2.3	27	90	0	4	1	0
27	41-50	1	2.4	29	90	15200	4	1	0
28	41-50	1	1.1	13	90	0	4	0	0
29	41-50	1	9.3	112	90	3010	3	0	0
30	41-50	0	1.8	21	90	0	4	0	0
31	41-50	0	2.1	25	90	634	4	1	0
32	41-50	1	5.3	64	90	0	4	1	0
33	41-50	1	4.3	51	90	295	3	0	0
34	41-50	1	3.6	43	70	183	4	0	0
35	41-50	1	1.1	13	90	43200	4	1	0
36	41-50	0	0.9	11	90	1600	4	0	0
37	41-50	1	0.8	9	90	0	3	0	0
38	41-50	1	2.7	32	90	3590	4	1	0
39	41-50	1	0.5	6	90	80700	3	0	0
40	41-50	1	2.5	30	90	0	4	1	0
41	41-50	1	1.6	19	90	0	3	1	0
42	41-50	1	15.2	182	90	0	3	1	0
43	41-50	1	1.2	14	80	0	4	0	0
44	41-50	1	1.1	13	90	183	4	0	0
45	41-50	1	4.3	51	90	0	3	0	0
46	41-50	1	1.3	15	90	0	3	1	0
47	41-50	1	1.8	22	70	0	3	0	0
48	41-50	1	1.8	22	90	0	4	1	0
49	41-50	1	3.4	41	90	183	3	0	0
50	41-50	1	1.7	20	90	606	3	0	0
51	41-50	1	1.5	18	90	18400	3	0	0

52	41-50	0	1.0	12	80	183	3	0	0
53	41-50	1	0.2	2	90	600	4	1	0
54	41-50	1	2.3	28	90	4420	4	1	0
55	41-50	0	2.1	25	90	0	3	1	0
56	41-50	1	10.2	122	90	0	3	0	0
57	41-50	1	2.0	24	90	0	3	0	0
58	41-50	1	1.7	20	90	183	3	0	0
59	41-50	1	1.5	18	90	0	3	0	0
60	41-50	1	5.6	67	90	0	3	0	0
61	41-50	1	4.2	50	90	26600	3	0	0
62	41-50	1	3.7	44	90	29700	3	0	0
63	51-60	1	2.3	28	90	12900	3	0	0
64	51-60	1	3.6	43	90	183	3	0	0
65	51-60	1	3.7	44	90	183	4	0	0
66	51-60	1	1.7	20	90	183	4	1	0
67	51-60	0	2.2	26	90	5660	4	0	0
68	51-60	1	2.7	32	60	183	4	0	0
69	51-60	0	3.2	38	90	0	4	0	0
70	51-60	0	6.4	77	90	192	3	0	0
71	51-60	1	1.0	12	90	183	4	0	0
72	51-60	0	1.6	19	70	4980	4	0	0
73	51-60	1	1.4	17	90	183	4	0	0
74	51-60	0	0.8	10	80	183	4	0	0
75	51-60	1	7.8	94	90	0	4	0	0
76	51-60	1	3.4	41	90	1840	3	0	0
77	51-60	0	1.2	14	90	183	3	0	0
78	51-60	1	2.3	27	80	183	4	0	0
79	51-60	1	10.1	121	90	46300	3	0	0
80	51-60	1	1.9	23	90	4070	3	0	0
81	51-60	1	3.3	39	90	0	3	0	0
82	51-60	0	1.3	16	90	183	3	0	0
83	51-60	1	1.8	22	90	1333	3	0	0
84	61-70	1	1.8	22	90	0	4	0	0
85	61-70	1	0.6	7	90	4680	4	1	0
86	61-70	0	1.4	17	90	0	4	0	0
87	61-70	1	1.8	21	80	5660	4	0	0
88	61-70	1	1.5	18	90	183	3	0	0

Significa	Reirradia	Dose of R	Volume	Mean dose	Max dose	Mini Dose	Fraction	Chemthera	Local Fai
0	1	64	62.8	63	67.2	58.9	29	1	1
0	0							1	1
1	0							1	1
1	1	64	53.8	64	71	57	29	1	1
0	1	64	113.7	66.9	70.1	62.8	28	1	0
0	0							1	1
0	1	64	48.9	70.1	73	67.2	33	1	1
0	0							1	0
0	0							1	1
0	1	64	87.5	68.7	71.7	65.9	32	1	0
0	1	64	28.3	68	72.3	64	32	1	1
0	1	66	55.1	68.7	71.9	65.3	33	1	0
0	0							1	0
1	0							1	0
1	1	60	58.3	62.5	66.1	59.2	30	1	0
1	1	64	48.1	68.3	71.7	59.9	32	1	0
0	1	60	33.2	63.9	66	61.8	28	0	1
0	0							1	1
0	0							1	1
1	1	64	44.2	66.6	71.7	61.4	33	1	0
1	0							1	0
0	0							1	1
1	1	64	45.3	67.8	69.8	65.9	32	1	0
0	1	60	35.3	62.6	66.6	58.2	27	1	0
1	1	64	103.8	68.8	74.9	62.7	32	1	0
1	1	64	56.1	69.1	73	65.3	32	1	1
0	1	60	15.2	64.6	66.8	61.6	30	1	0
0	1		59.5	66.7	71.3	64.5		1	0
0	1	62	69.3	65.4	72.8	57.8	31	1	0
0	0							1	1
0	0							1	1
0	0							1	0
0	0							1	0
1	1	66	26.1	67.7	70	65.3	32	0	0
0	1	63	79.1	63.5	64.9	62.4	30	1	0
0	0							1	1
0	0							1	1
0	0							1	0
1	1	64	59.2	67.8	72.2	65.5	32	1	0
1	1	68	59.1	72.4	76.2	69.4	30	1	1
1	0							1	1
0	0							1	1
0	1	64	27.9	65.9	72.3	59.5	30	1	0
1	1	60	57.5	63.2	66	60.6	30	1	1
0	1	60	89.5	61.6	66	57.6	27	1	0
0	1	60	121.8	60.7	64.8	56.4	27	0	0
1	1	66	28.7	69.7	72.5	67	29	0	1
1	1	66	39.3	64.7	70.6	58.7	30	1	0
0	0							1	0
0	1	64	21.3	66.3	68.3	64.4	30	1	1
0	1	64	63.7	66.4	71.3	56.8	28	1	1

0	1	60	48.4	62.9	65.9	57.4	28	1	0
0	0							1	1
0	0							1	1
1	1	70	26.6	74.9	79.1	70.7	35	0	0
0	1	60	81.5	63.7	66.6	56.4	32	1	0
0	0							1	0
0	0							1	0
0	0							1	0
0	0							1	0
0	0							1	0
0	0							1	0
1	1	70	26.3	77	80.5	73.5	35	1	1
1	1	64	47.7	66	68.9	62.1	30	1	0
1	1	58	59.3	58.6	61.5	55.7	30	1	1
0	0							1	0
0	0							1	0
1	0							1	1
1	1	66	45.2	68	71.3	64.7	33	0	0
0	1	70	44.7	72	77.2	72	35	1	0
0	0							1	0
1	0							1	1
1	1	64	62.2	65.7	72.3	58.9	32	1	0
0	0							1	1
0	0							1	0
0	0							1	0
1	1	64	81.6	63.6	67.2	59.5	32	1	0
1	1	60	32.3	60.8	70.9	53.9	27	0	1
0	0							1	0
1	1	64	57.9	65.6	68.5	62.7	32	1	0
1	1	66	40.8	69.8	74.6	65.3	32	1	0
0	0							1	1
0	0						30	1	1
0	1	65	61.7	67.3	71.6	63.4	30	1	0
0	0							1	0
0	0							1	0
0	0							1	0
0	0							1	0

Distant F	Progressi	Death	LFS	DMFS	TTP	OS
0	1	1	8	8	8	8
0	1	1	22	31	22	31
0	1	1	3	18	3	18
0	1	0	27	44	27	44
0	0	0	27	27	11	27
0	1	0	16	23	16	23
0	1	1	18	24	18	24
0	0	0	23	23	23	23
1	1	0	30	16	16	40
0	0	0	43	43	43	43
1	1	0	25	24	24	33
0	0	0	81	81	81	81
1	1	1	38	3	3	38
0	0	1	32	32	32	32
0	0	0	32	32	32	32
0	0	0	23	23	23	23
1	1	1	24	43	24	44
0	1	0	10	23	10	23
0	1	1	18	26	18	26
0	0	1	27	27	27	27
0	0	1	25	25	25	25
0	1	1	25	27	25	27
1	1	1	26	2	2	26
1	1	0	15	8	8	15
0	0	1	35	35	23	35
0	1	1	3	11	3	11
0	0	0	37	37	37	37
0	0	0	23	23	23	23
0	0	0	25	25	25	25
0	1	1	8	13	8	13
0	1	0	8	38	8	38
0	0	0	43	43	43	43
1	1	1	34	0	0	34
0	0	1	34	34	34	34
0	0	0	15	15	15	15
0	1	0	17	23	17	23
0	1	1	25	38	25	38
0	0	0	34	34	34	34
0	0	1	27	27	27	27
0	1	0	11	28	11	28
0	1	1	25	32	25	32
0	1	1	7	8	7	8
0	0	1	11	11	11	11
0	1	1	23	39	23	39
0	0	0	28	28	18	28
0	0	0	54	54	54	54
0	1	1	11	27	11	27
0	0	1	10	10	10	10
0	0	0	85	85	85	85
0	1	0	34	35	34	35
1	1	0	7	7	7	24

0	0	0	82	82	82	82
0	1	0	20	23	20	23
0	1	1	25	36	25	36
0	0	1	38	38	38	38
0	0	0	37	37	37	37
0	0	0	28	28	28	28
0	0	1	16	16	16	16
0	0	1	6	6	6	6
0	0	0	24	24	24	24
0	0	0	37	37	26	37
0	0	0	23	23	23	23
0	1	1	29	35	29	35
0	0	0	15	15	15	15
0	1	1	27	32	27	32
0	0	1	16	16	16	16
0	0	0	23	23	23	23
0	1	1	6	34	6	34
1	1	1	27	21	21	27
0	0	1	17	17	17	17
0	0	0	68	68	68	68
0	1	1	6	12	6	12
0	0	0	37	37	37	37
0	1	1	14	44	14	44
0	0	0	23	23	23	23
0	0	0	15	15	15	15
0	0	0	51	51	51	51
0	1	1	22	27	22	27
0	0	1	35	35	23	35
0	0	1	25	25	25	25
0	0	1	51	51	51	51
0	1	1	8	34	8	34
1	1	1	5	20	5	26
0	0	1	9	9	9	9
1	1	0	19	14	14	19
0	0	0	21	21	21	21
0	0	0	26	26	26	26
0	0	0	75	75	75	75
